# Emergency Medical Teams’ Responses during the West Japan Heavy Rain 2018: J-SPEED Data Analysis

**DOI:** 10.1017/S1049023X22000231

**Published:** 2022-04

**Authors:** Odgerel Chimed-Ochir, Yui Yumiya, Akihiro Taji, Eisaku Kishita, Hisayoshi Kondo, Akinori Wakai, Kouki Akahoshi, Kayoko Chishima, Yoshiki Toyokuni, Yuichi Koido, Tatsuhiko Kubo

**Affiliations:** 1. Department of Public Health and Health Policy, Graduate School of Biomedical and Health Sciences, Hiroshima University, Hiroshima, Japan; 2. Hiroshima Prefectural Health and Welfare Bureau, Hiroshima, Japan; 3. National Hospital Organization Headquarters DMAT Secretariat MHLW Japan, Tokyo, Japan; 4. National Hospital Organization Headquarters DMAT Secretariat MHLW Japan, Osaka City, Japan

**Keywords:** Emergency Medical Team, Emergency Medical Team minimum data set, Japan, J-SPEED, West Japan heavy rain

## Abstract

**Introduction:**

Rainfall-induced floods and landslides accounted for 20.7% of all disaster events in Japan from 1985 through 2018 and caused a variety of health problems, both directly and indirectly, including injuries, infectious diseases, exacerbation of pre-existing medical conditions, and psychological issues. More evidence of health problems caused by floods or heavy rain is needed to improve preparedness and preventive measures; however, collecting health data surrounding disaster events is a major challenge due to environmental hazards, logistical constraints, political and economic issues, difficulties in communication among stakeholders, and cultural barriers. In response to the West Japan Heavy Rain in July 2018, Emergency Medical Teams (EMTs) used Japan - Surveillance in Post-Extreme Emergencies and Disasters (J-SPEED) as a daily reporting template, collecting data on the number and type of patients they treated and sending it to an EMT coordination cell (EMTCC) during the response.

**Study Objective:**

The aim of the study was to conduct a descriptive epidemiology study using J-SPEED data to better understand the health problems during floods and heavy rain disasters.

**Methods:**

The number and types of health problems treated by EMTs in accordance with the J-SPEED (Ver 1.0) form were reported daily by 85 EMTs to an EMTCC, where data were compiled during the West Japan Heavy Rain from July 8 through September 11, 2018. Reported items in the J-SPEED form were analyzed by age, gender, area (prefecture), and time period.

**Results:**

The analysis of J-SPEED data from the West Japan Heavy Rain 2018 revealed the characteristics of a total of 3,617 consultations with the highest number of consultations (2,579; 71.3%) occurring between Day 5 and Day 12 of the 65-day EMT response. During the response period, skin disease was the most frequently reported health event (17.3%), followed by wounds (14.3%), disaster stress-related symptoms (10.0%), conjunctivitis (6.3%), and acute respiratory infections (ARI; 5.4%).

**Conclusion:**

During the response period, skin disease was the most frequently reported health event, followed by wounds, stress, conjunctivitis, and ARIs. The health impacts of a natural disaster are determined by a variety of factors, and the current study’s findings are highly context dependent; however, it is expected that as more data are gathered, the consistency of finding will increase.

## Introduction

The world has witnessed an increase in disaster events over the past several decades. Floods account for 40%-50% of all disasters and disaster-related deaths world-wide^1,2^ and have widespread social and health impacts. Rainfall-induced floods and landslides accounted for 20.7% of all disasters in Japan from 1985 through 2018,^3^ and annual rainfall of 50mm per hour has increased 1.4-times in the last 30 years, making flood prevention a crucial issue.^4^ Flood causes a variety of health problems, both directly and indirectly, including injuries, infectious diseases, exacerbation of pre-existing medical conditions, and psychological issues.^5,6^ More evidence of health problems caused by floods or heavy rain is needed to improve preparedness and preventive measures;^7^ however, collecting health data surrounding disaster events is a major challenge due to environmental hazards, logistical constraints, political and economic issues, difficulties in communication among stakeholders, and cultural barriers.^8^

The Emergency Medical Team (EMT) is a group of health professionals that includes doctors, nurses, paramedics, support workers, and logisticians who treat patients affected by an emergency or disaster. These EMTs provide direct clinical care to disaster victims and play a major role in the health care support system during a disaster. Observations from the Great East Japan Earthquake in 2011included a need for standardized EMT daily reporting. The Joint Committee for Disaster Medical Recording was established and proposed the standard disaster medical and daily reporting forms called the Japan - Surveillance in Post-Extreme Emergencies and Disasters (J-SPEED; Committee Report 2015). The J-SPEED is based on the Surveillance in Post-Extreme Emergencies and Disasters (SPEED) of the Republic of the Philippines and allows EMTs in Japan to collect real-time health data during emergencies and disasters.^9,10^

The West Japan Heavy Rain in July 2018 triggered a major activation of EMTs in Japan. All EMTs used the J-SPEED as a daily reporting template, collecting data on the number and type of patients they treated and sending it to the EMT coordination cell (EMTCC) during the response. The aim was a descriptive epidemiology study using J-SPEED data to better understand the health problems during floods and heavy rain disasters.

## Methods

### Study Design

This is the descriptive analysis of daily reports collected by EMTs during the West Japan Heavy Rain that occurred from July 8 through September 11, 2018.

### Data Collection

The number and types of health problems treated by EMTs in accordance with the J-SPEED (Ver 1.0) form were reported on a daily basis by EMTs to the EMTCC, where data were compiled during the West Japan Heavy Rain from July 8 through September 11, 2018.

On the J-SPEED (Ver1.0) form, 26 items are defined and suggested to be reported, including demographic information and health events that were major and relevant to EMT operation. Furthermore, there were four additional blank items that would be defined by EMTCC during disaster response based on the disaster’s specific context. Heat stroke and conjunctivitis were suggested by EMTCC and reported by EMTs as additional items during the West Japan Heavy Rain. The items examined in this study are shown in a Supplemental Table (available online only).

The J-SPEED form was used by 85 EMTs who were dispatched to three prefectures: Hiroshima, Okayama, and Ehime. On July 8, EMTs started and reported clinical services in Okayama and Hiroshima Prefectures, and on July 17, they started in Ehime Prefecture. A total of 3,620 consultations were reported by 85 EMTs. Only three deaths were reported among them. After excluding the deaths, 3,617 consultations remained for analysis in the current study.

### Data Analysis

All items reported by the J-SPEED form were analyzed by age, gender, area (prefecture), and time period. The J-SPEED form was available in two formats, paper and mobile application. Earlier in the response, the J-SPEED paper form was used for daily reporting, but the J-SPEED mobile application was used concurrently during the latter part of the response. The paper form had age groups of 0 years, 1-8, 9-74, and 75+ whereas the mobile application had age groups of 0 years, 1-14, 15-64, and 65+. To merge the age categories of the two J-SPEED formats, age groups of 0 years, 1-14, 15-64, and 65+ were converted to 0 years, 1-8, 9-74, and 75+. In addition, age groups 0 years and 1-8 were combined because the number of health problems attributed to 0-year-old patients only accounted for 0.5% (N = 17) of total health events. By referring to the peak and trend of the reported number of patients, the time period was divided into five periods (Days 1-4, Days 5-8, Days 9-12, Days 13-16, and Day 17 or later). Microsoft Excel (Microsoft Corp.; Redmond, Washington USA) and STATA v15.1 (STATA Corp; College Station, Texas USA) were used for analysis.

### Ethical Consideration

Approval for ethical review was obtained from Hiroshima University (Hiroshima, Japan; approval number: E-2059). This study was funded by the World Health Organization Kobe Centre for Health Development (WKC-HEDRM-K19009), Grants for Research on Policy Planning and Evaluation from the Ministry of Health, Labor, and Welfare, Japan (grant numbers:19IA2014, 20CA2063), and JSPS KAKENHI Grant Number JP21K09020.

## Results

**Figure 1.**
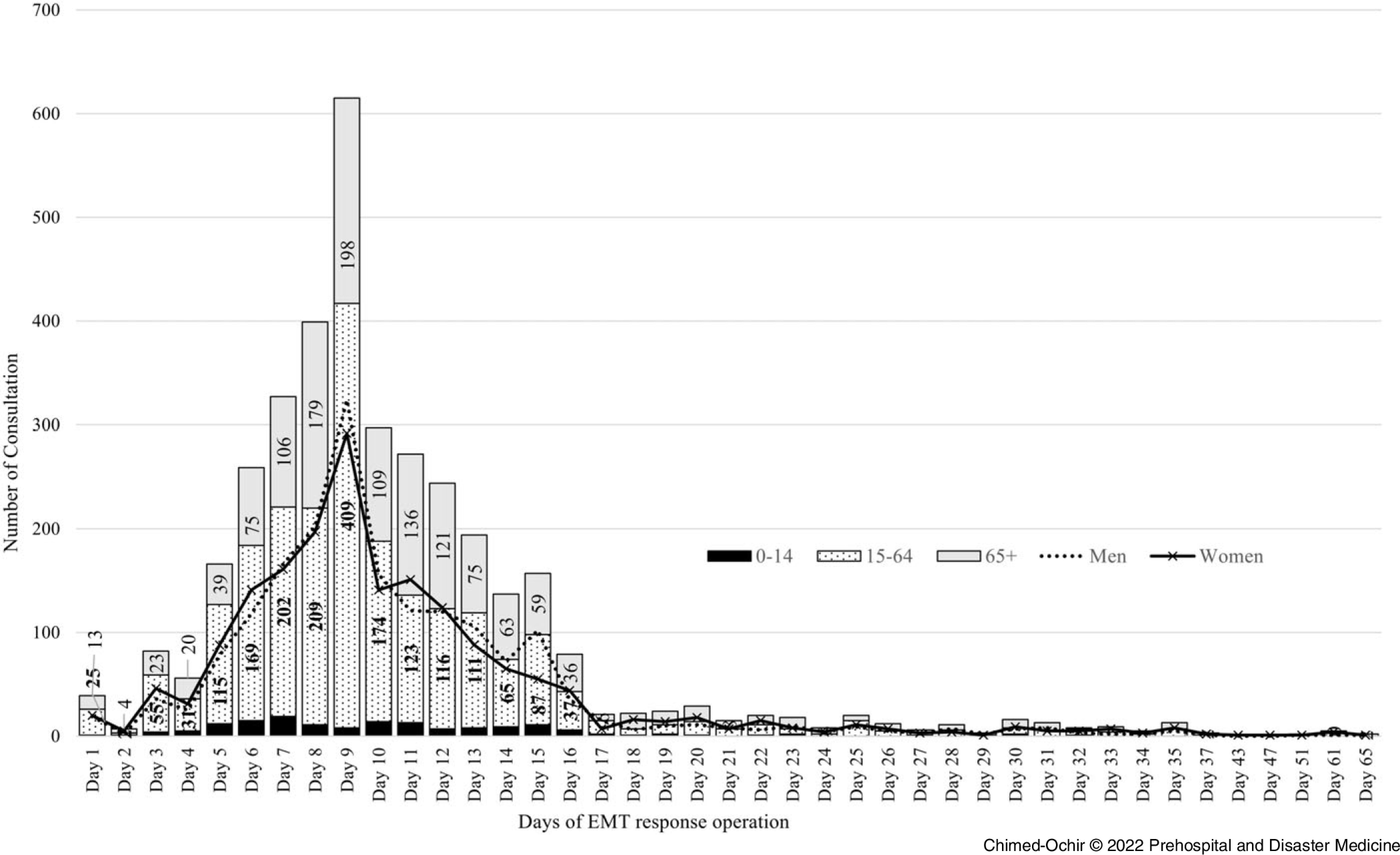
Distribution of Consultations by Emergency Medical Teams during the West Japan Heavy Rain, 2018. Abbreviation: EMT, Emergency Medical Team.

Figure 1 shows the number of consultations by sex, age, and day of EMT operations. Initially, the number of consultations increased drastically, peaking on Day 9 with 607 consultations (16.8% of total consultations), then decreased until Day 17, after which reporting was consistent through Day 65.

Table 1 shows the location of consultations provided by EMTs. A total of 3,617 consultations were reported with the majority (71.3%) being provided between Day 5 and Day 12. Medical consultations were distributed across Okayama (64.1%), Hiroshima (34.8%), and Ehime (1.1%) prefectures with an equal distribution between men (50.0%) and women (50.0%; Table 2).

**Table 1.** Location of Consultations Provided by Emergency Medical Teams during the West Japan Heavy Rain, 2018

Day			Prefecture					
	Total		Okayama		Hiroshima		Ehime	
	N	%	N	%	N	%	N	%
Day 1-4	184	5.1%	111	3.1%	73	2.0%	–	–
Day 5-8	1,151	31.8%	792	21.9%	359	9.9%	–	–
Day 9-12	1,428	39.5%	989	27.3%	435	12.0%	4	0.1%
Day 13-16	567	15.7%	428	11.8%	135	3.7%	4	0.1%
Day 17+	287	7.9%	–	0.0%	257	7.1%	30	0.8%
**Total**	**3,617**	100.0%	**2,320**	64.1%	**1,259**	34.8%	**38**	1.1%

**Table 2.** Demographic Information of Consultations by Emergency Medical Teams during the West Japan Heavy Rain, 2018

Day	Total	Sex				Age					
		Men		Women		0-8		9-74		75+	
	N	N	%	N	%	N	%	N	%	N	%
Day 1-4	184	82	2.3%	102	2.8%	11	0.3%	113	3.1%	60	1.7%
Day 5-8	1,151	564	15.6%	587	16.2%	57	1.6%	695	19.2%	399	11.0%
Day 9-12	1,428	720	19.9%	708	19.6%	42	1.2%	822	22.7%	564	15.6%
Day 13-16	567	315	8.7%	252	7.0%	34	0.9%	300	8.3%	233	6.4%
Day 17+	287	126	3.5%	161	4.5%	12	0.3%	132	3.6%	143	4.0%
**Total**	**3,617**	**1,807**	50.0%	**1,810**	50.0%	**156**	4.3%	**2,062**	57.0%	**1,399**	38.7%

Table 2 also shows the age distribution of consultations, and patients under the age of 14 made up 4.3% (156), ages 15 to 64 made up 57% (2062), and those 65 and over received 38.7% (1399) of the consultations.

Table 3 shows the health events reported by EMTs. Out of 3,617 consultations recorded, skin disease accounted for the highest percentage (627; 17.3%) followed by wounds (518; 14.3%), non-disaster-related events (383; 10.6%), disaster stress-related symptoms (361; 10.0%), conjunctivitis (228; 6.3%), acute respiratory infections ([ARI] 195; 5.4%), and heatstroke (183; 5.1%).

**Table 3.** Health Events Reported by Emergency Medical Teams during the West Japan Heavy Rain, 2018

	Health Events/Age Group (years old)	Total		0-8		9-74		75+	
		N	%	N	%	N	%	N	%
	**Total Number of Consultations**	**3,617**	(100.0%)	**156**	(4.3%)	**2,062**	(57.0%)	**1,399**	(38.7%)
	**Health Events**								
1	Skin Diseases	627	[17.3%]	49	[31.4%]	399	[19.4%]	179	[12.8%]
2	Wound	518	[14.3%]	10	[6.4%]	350	[17.0%]	158	[11.3%]
3	Disaster Stress-Related Symptoms	361	[10.0%]	6	[3.8%]	200	[9.7%]	155	[11.1%]
4	Conjunctivitis	228	[6.3%]	7	[4.5%]	131	[6.4%]	90	[6.4%]
5	Acute Respiratory Infections	195	[5.4%]	20	[12.8%]	91	[4.4%]	84	[6.0%]
6	Hypertension	183	[5.1%]	1	[0.6%]	85	[4.1%]	97	[6.9%]
7	Heat Stroke	183	[5.1%]	5	[3.2%]	138	[6.7%]	40	[2.9%]
8	Fever	62	[1.7%]	11	[7.1%]	29	[1.4%]	22	[1.6%]
9	Gastrointestinal Infections	59	[1.6%]	5	[3.2%]	30	[1.5%]	24	[1.7%]
10	Deep Vein Thrombosis	30	[0.8%]	0	[0.0%]	13	[0.6%]	17	[1.2%]
11	Bronchial Asthma	30	[0.8%]	2	[1.3%]	21	[1.0%]	7	[0.5%]
12	Fracture	21	[0.6%]	0	[0.0%]	13	[0.6%]	8	[0.6%]
13	Burn	13	[0.4%]	1	[0.6%]	10	[0.5%]	2	[0.1%]
14	Urgent Need for Psychological Support	11	[0.3%]	0	[0.0%]	5	[0.2%]	6	[0.4%]
15	Suspected Tetanus	10	[0.3%]	0	[0.0%]	4	[0.2%]	6	[0.4%]
	**Other Events**								
16	Interrupted Essential Medications	150	[4.6%]	1	[0.6%]	62	[3.0%]	87	[6.2%]
17	Needs for Transfer	23	[0.7%]	0	[0.0%]	12	[0.6%]	11	[0.8%]
18	Triage	18	[0.5%]	1	[0.6%]	13	[0.6%]	4	[0.3%]
19	Non-Disaster-Related Events	383	[10.6%]	16	[10.3%]	139	[6.7%]	228	[16.3%]

Among the age groups, the prevalence of skin diseases, ARIs, fever, gastrointestinal infections, bronchial asthma, and burn was highest among children (0-8 years old); wound, disaster stress-related symptoms, and heatstroke were highest among adult patients (9-74 years old); hypertension, deep vein thrombosis, urgent need for psychological support, and interrupted essential medications, needs of transfer, and non-disaster-related events were highest among elderly patients (over 75 years old).

**Figure 2.**
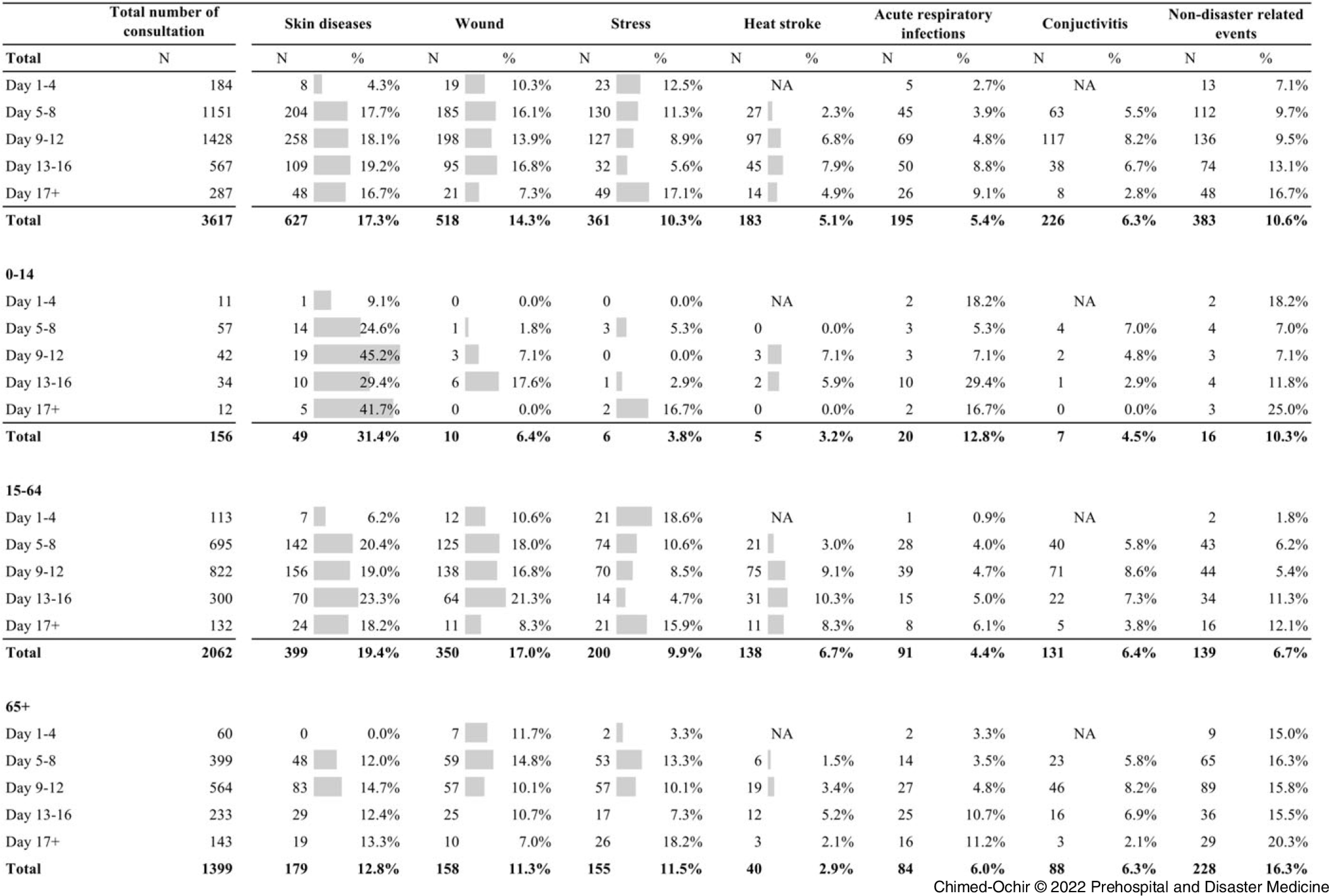
Major Health Events by Period and Age Groups Reported by Emergency Medical Teams during the West Japan Heavy Rain, 2018.

Figure 2 shows the major health events by patient age groups and period of EMT operation. The major health events included the four health problems of highest prevalence (skin diseases, wound, disaster stress-related symptoms, and ARIs); two additional symptoms (heatstroke and conjunctivitis); and non-disaster-related events. Focusing on specific health problems requiring consultations over time, the proportion of skin diseases increased soon after the EMTs began operating, most visibly for children. Wounds, heat stroke, and conjunctivitis had visible peaks in terms of their proportion to total events in corresponding time periods. Also, adults recorded a higher proportion of wounds throughout the response period. Disaster stress-related symptoms, ARIs, and non-disaster-related events peaked at the end of the response period. Throughout the entire period of response, a high proportion of non-disaster-related events were observed among elderly patients.

## Discussions

The analysis of J-SPEED data from the West Japan Heavy Rain 2018 disaster reported by 85 EMTs revealed the characteristics of a total of 3,617 consultations with a majority of consultations (2,579; 71.3%) occurring between Day 5 and Day 12 of the EMT response. During the response period, skin diseases were the most frequently reported health event, followed by wounds, disaster stress-related symptoms, conjunctivitis, and ARIs.

In the field of emergency medical response and natural disasters, the existing literature uses data and information collected from a variety of sources, including local hospital records, interviews with victims, routine death certificates, emergency clinical data, and other retrospective surveys.^11^ The J-SPEED data used in this study were collected in real time by operating EMTs during the disaster response, which is very unique for this study. In other words, the current study’s findings are highly context dependent.

In addition, health impacts of a natural disaster depend not only on the type and scale of the disaster, but also on a variety of factors such as geography and weather conditions, patterns of exposure, community infrastructure including hygiene setting, emergency preparedness, underlying vulnerability, and culture of the population.

This analysis includes the available global evidence while taking Japan-specific characteristics into account. The results can in turn identify good practice and lessons learned not only for Japan, but for other countries as well.

In the current analysis, it was discovered that skin diseases accounted for nearly one-third of all consultations. This is consistent with previous studies which found that inflammatory skin diseases, skin infections, traumatic skin diseases, insect bite reactions, and psycho-emotional aggravated primary skin diseases were common problems during floods.^12,13^ Although the J-SPEED data don’t allow to specify the exact type of skin diseases, it can still be suggested that skin disease is a common health problem not only in developing countries, but also in developed countries during heavy rain disasters. The current findings also revealed that skin disease is most common among children aged 0-8 years. Some literature reported no significant association between age and skin problems,^14^ while others found that older patients were more likely to have skin diseases than younger adults.^15^ Thus, a consensus for this issue could not be found. There was a contextual episode identified regarding skin disease and conjunctivitis during the West Japan Heavy Rain. Following an increase in the number of reports of skin disorders and conjunctivitis, dermatologists and ophthalmologists participated in EMT services in the field and diagnosed the majority of those cases as caused by lime sprayed for disinfection. As a result, the EMTCC, in collaboration with local public health authorities, advised the public to wear appropriate protective gear to protect their skin and eyes.

Wounds and heat stroke were more common among those aged 9 to 74 years in this disaster response. Previous studies noted high incidence of wound due to flood,^16^ however, the reasons for wounds vary, such as collapse of buildings or other structures or fast-moving water containing debris.^16^ Garbern, et al^17^ revealed in their review study that heat-related problems are common during disaster in a hot environment. In the case of West Japan Heavy Rain, adults were heavily engaged in outdoor activities such as cleaning debris and reconstructing housing in an extremely hot environment. This context may help to explain the increased number of injuries and heat strokes among the adults during the mid-phase of the response. Heat acclimatization among the evacuees and the installation of spot coolers in shelters may be attributed to the decrease in number and proportion of heat strokes during the latter phase. Avoidable environmental problems, such as heatstroke, need to be well-managed through preventable measures, and during the West Japan Heavy Rain response, the EMTCC continuously announced preventable measures for heat stroke based on the number of cases reported during the response. Previous research has shown that children and elderly are particularly vulnerable to adverse physical consequences during floods;^18^ however, it was discovered that the working-age population was more vulnerable to most of the reported diseases, with the exception of blood pressure and thrombosis. As previously stated, this could be attributed to their active participation in some activities such as cleaning home during or after disaster.

It was found that ARIs were one of the most common problems among people of all ages. This is consistent with other studies that found ARIs to be the most common type of infectious disease among both developed^1,19^ and developing countries.^20^ In the current context, elders and children who stayed in shelters may have been affected by crowding which may have increased ARIs.

Unlike ARI, diarrheal diseases accounted for only 1.6% of total consultations in the current study. It is evident that the risk of gastrointestinal infection after flooding is highest, especially in developing countries, in areas with compromised hygiene practices, an inadequate supply of clean drinking water, unavailability of clean water for washing, inaccessibility of toilet,^21^ and over-crowding.^22,23^ Some local practices such as fecal sludge application in agricultural communities,^24^ the conditions to promote the bacterial transmission via fecal-oral route by ingestion of contaminated food or water in developing countries,^25,26^ are reported to be the contributing factors to the water-borne diseases during floods. The risk of outbreaks of diarrheal diseases is reported as low in high-income countries, however, it increases if the integrity of sewerage systems is compromised or unhygienic conditions arise.^27,28^ Japan is known for having a high standard of cleanliness and hygiene and local governments provided sufficient amounts of clean drinking water during the disaster. In addition, the recovery time for disrupted water supply was relatively short. For example, during the Great East Japan Earthquake of 2011, the most tragic disaster in Japanese history, the median time of service recovery was three days.^29^ Therefore, the low incidence of water-borne diseases during disaster in Japan may have been due to a high standard of hygiene, availability of drinking water supplies, and good personal hygiene.

Disaster-related stress symptoms were another major health problem. This is in line with innumerable studies that have found that disasters greatly increase susceptibility not only to physical illness, but also to poor mental health.^30–33^ The current study showed that the incidence rate of stress was high during the early days and peaked in the later days. Stress may have derived from the panic caused by the sudden onset of events in the early days, as well as physical health issues, personnel losses, and economic hardship in the later days. The EMTs should be aware of physical conditions, socioeconomic status, and any other triggering factors throughout the response period.

Exacerbation of chronic diseases is one of the most common reasons for presenting to emergency facilities during^34^ natural disasters. The EMT received 150 patients (4.6% of total consultation; Table 2), with more than one-half being elderly people, with their treatment interrupted. Therefore, treatment disruptions may have contributed to exacerbation of chronic conditions in the elderly. Treatment disruption may occur as a result of flood damage to health facilities or inaccessibility to health facilities during heavy rain as has been seen in flooding even in developed countries.^35^ Thus, in light of the growing aged populations, it is critical to ensure that patients with chronic conditions are monitored and their medication is maintained during floods.^36^

Non-disaster-related events accounted for 10% of total consultations, with elders accounting for more than one-half of them. Distinguishing between disaster-related and non-related events is not a simple task, and for the J-SPEED reporting, judgment is left to the medical doctors of EMTs examining patients. Based on this practical reporting, it was discovered that non-disaster-related events increased as time passed after the disaster. During the response, the transition of this characteristic was used as an indicator to guide and support the EMTCC’s decision to demobilize the EMTs.

## Strengths and Limitations

There are some strengths and limitations of this study worth mentioning. The main strength is that J-SPEED, which was used in the data collection here, is a new standard data reporting system among Japanese EMTs. Real-time data reporting enables the EMTCC to conduct data-driven coordination during the response, as well as epidemiological studies which include contextual data and enhances application of lessons learned.

In regard of limitations, J-SPEED reporting was initiated from EMTs rather than local health facilities, so the data did not cover overall clinical activities. For example, no case of drowning, which is one of the leading causes of morbidity due to flood,^36^ was reported by EMTs in this study; however, it cannot be concluded that the risk of drowning was low because cases could have been transported directly to local hospitals. Because of these reporting characteristics, relevant health risks may be under-estimated. Second, because J-SPEED reporting is still a new procedure, the reporting and data collection process during in-field emergency response faces a number of challenges, including a lack of pre-training and a lack of understanding of the definitions of reporting items and J-SPEED reporting procedures. The collected data were “quick and dirty” and missing data or unrecorded patients were a common issue during data collection with J-SPEED. As a result, under-reporting occurred resulting in an under-estimation of each health event. Third, because the age groups used in the J-SPEED paper form and the J-SPEED smartphone app were merged (as described in the Methods section), there may be some bias in the distribution of health events for age groups. However, because the age distribution trend did not show a significant gap during the period, the impact may have been limited.

## Conclusion

The analysis of J-SPEED data from the West Japan Heavy Rain 2018 reported by 85 EMTs provides a detailed characterization of 3,617 consultations. During the response period, skin disease was the most frequently reported health event, followed by wounds, stress, conjunctivitis, and ARIs. The health impacts of natural disaster are determined by a variety of factors, and the current study’s findings are highly context dependent; however, it is expected that as more data are gathered in future studies, the consistency of findings will increase.

## Supplementary material

For supplementary material accompanying this paper visit https://doi.org/10.1017/S1049023X22000231. click here to view supplementary material
