# Supplementary figures and images for: Emergency Medical Teams’ Responses during the West Japan Heavy Rain 2018: J-SPEED Data Analysis
Source: Prehosp Disaster Med. 2022 Feb 28;37(2):205–11. doi: 10.1017/S1049023X22000231 (PMC8958047; doi:10.1017/S1049023X22000231)

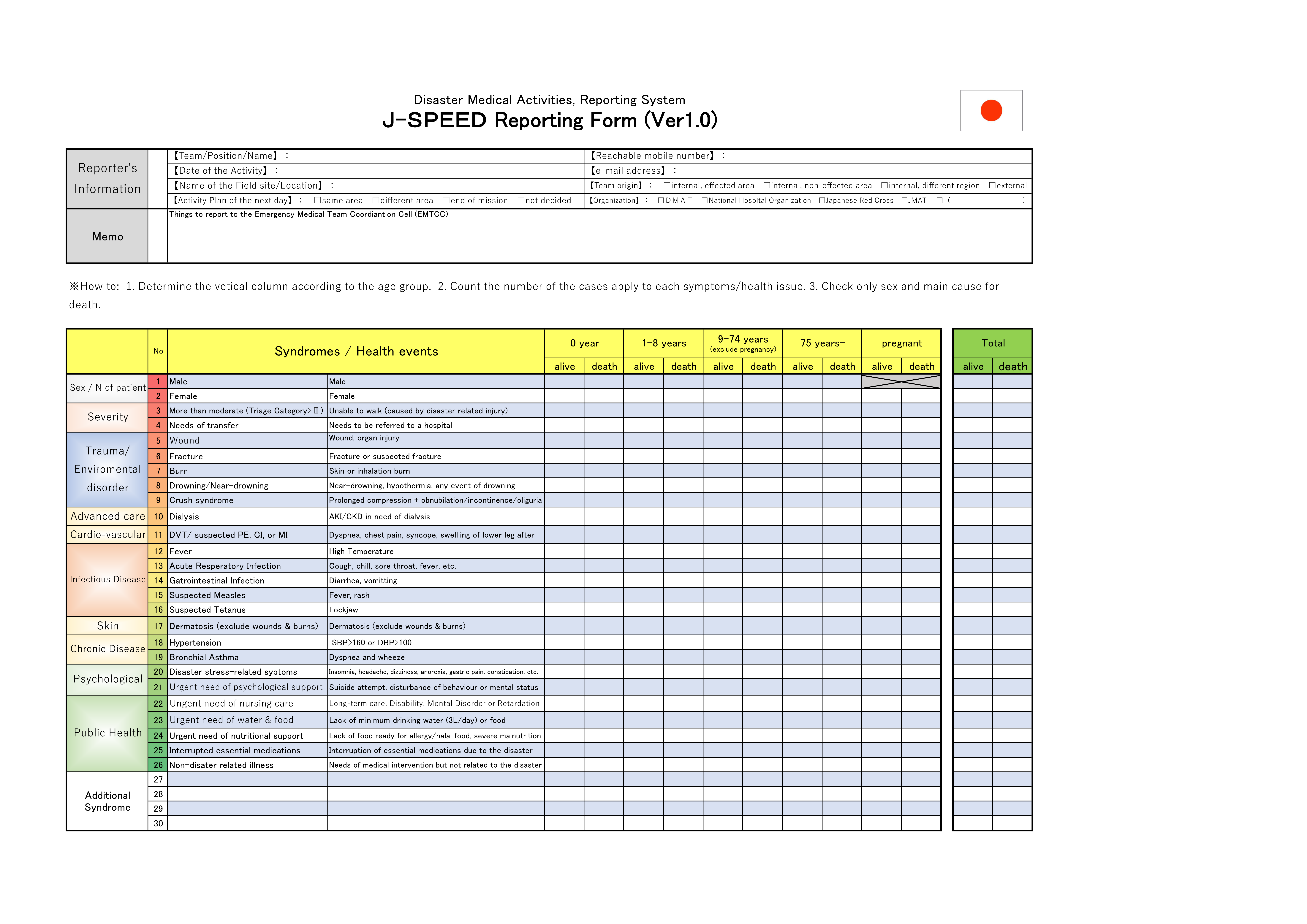

Supplement: Supplementary file 1 [file S1049023X22000231sup001.tif]
